# A statistical modelling approach for source attribution meta‐analysis of sporadic infection with foodborne pathogens

**DOI:** 10.1111/zph.12937

**Published:** 2022-03-10

**Authors:** Lapo Mughini‐Gras, Elisa Benincà, Scott A. McDonald, Aarieke de Jong, Jurgen Chardon, Eric Evers, Axel A. Bonačić Marinović

**Affiliations:** ^1^ Centre for Infectious Disease Control (CIb) National Institute for Public Health and the Environment (RIVM) Bilthoven The Netherlands; ^2^ Institute for Risk Assessment Sciences (IRAS), Faculty of Veterinary Medicine Utrecht University Utrecht The Netherlands; ^3^ Office for Risk Assessment & Research (BuRO) Netherlands Food and Consumer Product Safety Authority Utrecht The Netherlands

## Abstract

Numerous source attribution studies for foodborne pathogens based on epidemiological and microbiological methods are available. These studies provide empirical data for modelling frameworks that synthetize the quantitative evidence at our disposal and reduce reliance on expert elicitations. Here, we develop a statistical model within a Bayesian estimation framework to integrate attribution estimates from expert elicitations with estimates from microbial subtyping and case‐control studies for sporadic infections with four major bacterial zoonotic pathogens in the Netherlands (*Campylobacter*, *Salmonella*, Shiga toxin‐producing *E. coli* [STEC] O157 and *Listeria*). For each pathogen, we pooled the published fractions of human cases attributable to each animal reservoir from the microbial subtyping studies, accounting for the uncertainty arising from the different typing methods, attribution models, and year(s) of data collection. We then combined the population attributable fractions (PAFs) from the case‐control studies according to five transmission pathways (domestic food, environment, direct animal contact, human–human transmission and travel) and 11 groups within the foodborne pathway (beef/lamb, pork, poultry meat, eggs, dairy, fish/shellfish, fruit/vegetables, beverages, grains, composite foods and food handlers/vermin). The attribution estimates were biologically plausible, allowing the human cases to be attributed in several ways according to reservoirs, transmission pathways and food groups. All pathogens were predominantly foodborne, with *Campylobacter* being mostly attributable to the chicken reservoir, *Salmonella* to pigs (albeit closely followed by layers), and *Listeria* and STEC O157 to cattle. Food‐wise, the attributions reflected those at the reservoir level in terms of ranking. We provided a modelling solution to reach consensus attribution estimates reflecting the empirical evidence in the literature that is particularly useful for policy‐making and is extensible to other pathogens and domains.

**Impacts**

- A statistical model within a Bayesian estimation framework was developed to combine attribution estimates from expert elicitations with estimates from microbial subtyping and case‐control studies of sporadic infection with zoonotic pathogens to reach consensus estimates reflecting the available empirical evidence.
- Attribution estimates were robust and biologically plausible and allowed human cases to be attributed in several ways according to reservoirs, transmission pathways and food groups, which is particularly useful for policy‐making.
- The four pathogens attributed were all predominantly foodborne, with *Campylobacter* mostly attributable to chicken, *Salmonella* to pigs, *Listeria* and STEC O157 to cattle. Food‐wise, the attributions reflected those at the reservoir level.

## 1 INTRODUCTION

It is estimated that, annually, bacterial foodborne infections cause ~350 million illnesses and ~187 thousand deaths globally, accounting for ~14.5 million disability‐adjusted life years (DALY) (WHO, 2015). Food reaches the consumer through complex production chains in which many opportunities for (re‐)contamination exist. For policy‐making, it is crucial to know which fraction of the disease burden is attributable to food and which food products contribute to that fraction. To this end, source attribution is used. Source attribution is defined as the partitioning of human disease burden (DALY, incidence, costs, etc.) for a given pathogen over their animal, food, environmental and human (if any) sources of infection (Pires et al., 2009).

The umbrella term ‘source attribution’ consists of several methods and data types, a detailed overview of which is available elsewhere (Mughini‐Gras et al., 2019; Pires et al., 2009). Briefly, source attribution approaches can be divided into ‘top‐down’ and ‘bottom‐up’. Top‐down approaches attribute the human cases (i.e. the ‘top’ of the transmission chain) back to their sources of infection (i.e. the ‘bottom’), and these approaches can be further divided into epidemiological methods, e.g. analysis of outbreak investigations (Pires et al., 2010, 2019) and case‐control/cohort studies (Domingues et al., 2012a, 2012b), and microbiological methods (i.e. microbial subtyping (Barco et al., 2013)), or a combination of both (Mossong et al., 2016; Mughini Gras et al., 2012; Mughini‐Gras, Enserink, et al., 2014; Mughini‐Gras et al., 2018; Rosner et al., 2017). Conversely, bottom‐up approaches like comparative exposure assessment (Pintar et al., 2016) aim to predict the number of human cases caused by a given pathogen in a source, starting from the level of contamination (prevalence and concentration) and then incorporating effects of food processing, preparation, storage and consumption, and dose–response relationships.

When data are scarce, source attribution is often done using structured expert elicitations (Havelaar et al., 2008). Expert elicitations, however, can only provide an indication of consensus opinions among (some) experts in the field. Several limitations undermine the reliability of expert elicitations, such as the issues of properly accounting for qualitative arguments, cognitive heuristics and overconfidence, the selection/representativeness of experts’ backgrounds and agendas, and the uncertainty and diversity in opinions. Although expert elicitations remain a highly valuable instrument for policy‐making, they should be considered a low‐cost/low‐effort alternative to the generation, analysis and interpretation of empirical data. Expert elicitations should actually build on these data and be undertaken only when knowledge remains inadequate or conflicting (Morgan, 2014). Some authors have therefore called for novel data‐driven approaches to source attribution to combine different types of data in a single analytical framework (Havelaar et al., 2008; Koutsoumanis et al., 2019; Pires et al., 2018).

For several foodborne pathogens, numerous source attribution studies based on epidemiological and microbiological methods have been performed (Mughini‐Gras et al., 2019). These studies provide empirical data for input into a modelling framework that synthesizes the quantitative evidence at our disposal and constrains it with the estimates from expert elicitations as to make best use of all available evidence. In the Netherlands, expert elicitations are routinely used to attribute the burden of 17 enteropathogens to five major transmission pathways (food, environment, direct animal contact, human–human transmission and travel) and 11 groups within the food pathway (beef/lamb, pork, poultry meat, eggs, dairy, fish/shellfish, fruit/vegetables, beverages, grains, composite foods and food handlers/vermin), but the elicitation in question was conducted in 2008 and has therefore become outdated (Havelaar et al., 2008). Here, we present a way to integrate the existing expert estimates with available empirical data derived from source attribution analyses based on microbial subtyping and case‐control studies of sporadic foodborne infection. To this goal, we developed a quantitative statistical modelling approach and applied it to four major bacterial zoonotic pathogens in the Netherlands (i.e. *Campylobacter*, *Salmonella*, *Shiga* toxin‐producing *E. coli* [STEC] O157 and *Listeria monocytogenes*), although the approach can in principle be applied to other pathogens and domains as well.

## 2 METHODS

### 2.1 Data collection

A literature search was conducted in January 2019 using a combination of keywords related to (a) the foodborne pathogen (e.g. ‘Shiga* *Escherichia coli* OR Shiga* *E coli* OR STEC OR VTEC etc.’ or ‘*Salmonella* OR salmonellosis’ etc.) and (b) a general term indicating the type of study ‘source attribution OR microbial subtyping OR case‐control study’, joined by the logical connector AND, using Google Scholar, Science Direct, PubMed, Scielo, ISI Web of Science and Scopus. A similar search strategy has been used in a recent collection of manuscripts on systematic reviews and meta‐analyses of risk factors for 11 sporadic foodborne diseases globally (Gonzales‐Barron et al., 2021). There were no restrictions in terms of year and type of publication. The search was limited to the languages English and Dutch. Each reference was manually and independently screened for relevance by two reviewers, and the following inclusion criteria were applied: the data reported in the study had to be collected from the year 2000 onwards, and the study had to be performed in the Netherlands. If there was no Dutch study available, the inclusion criterion was relaxed to include also studies performed outside the Netherlands. For this, the global overview of case‐control studies provided by the aforementioned collection of systematic reviews and meta‐analyses (Gonzales‐Barron et al., 2021), and other recent review papers (Koutsoumanis et al., 2019; Mughini‐Gras et al., 2019) were also used.

The data extracted from the studies based on microbial subtyping that passed the screening were pathogen, typing method, year(s) of data collection, country of the study, source attribution model, number of human cases attributed, sources which the human cases were attributed to and the per cent attribution estimates (the attribution estimates provided by these studies, by default, sum up to 100% over the sources). For the case‐control studies, data extraction included the study characteristics (i.e. year, country, population studied and sample size) the statistical analysis performed, the statistically significant risk factors and the outcomes of the study (odds ratios [ORs] and, if available, the corresponding population attributable fractions [PAFs]). Only case‐control studies of sporadic cases (i.e. not those conducted in outbreak settings) performed in the Netherlands from 2000 onwards using multivariable logistic regression and reporting ORs as a measure of association were considered. Following standard epidemiological terminology, sporadic cases are those occurring irregularly/occasionally in time and place (i.e. scattered or isolated) and differ from the outbreak‐related cases because they cannot be linked to one another or to a common source of infection. The use of adjusted ORs was done to account for possible confounding that needed to be controlled for within each study through multivariable analysis. All studies included in the analysis were checked for redundancy, i.e. whether they used the same data and methods. The focus was on sporadic cases for different reasons: (a) sporadic cases are the majority of cases and are generally more difficult to attribute to sources, as they are not linked to one another or to a common source of infection (Domingues et al., 2012a, 2012b); (b) outbreaks are usually caused by one (or just a few) pathogen strain (as clonality is common within an outbreak) and one (or just a few) exposure; (c) outbreak data are usually synthetized using a specific type of meta‐analysis (Pires et al., 2010, 2019); (d) combining attributions for sporadic and outbreak‐related cases are not recommended, as outbreaks tend to be investigated disproportionally more often than sporadic cases. The epidemiology of a same pathogen can be quite different when this pathogen appears sporadically or as part of an outbreak, with some sources being disproportionally represented among outbreak‐related versus sporadic cases simply because these sources (e.g. drinking water) are more likely to be identified and have the potential to cause larger, albeit extremely rare, outbreaks (Pires et al., 2010).

### 2.2 Analytical approach

For the four pathogens included here, there are different transmission routes from an animal reservoir. For example, if the reservoir is a food‐producing animal, transmission can be foodborne, but also environment‐mediated or occurring through direct contact with the animal. Source attribution based on microbial subtyping usually attributes human cases at the level of reservoir (or amplifying host) regardless of the transmission pathway, whereas case‐control studies usually provide attributions at the exposure level (Mughini‐Gras et al., 2019; Pires et al., 2009). To address these methodological differences while providing attributions comparable with those based on the aforementioned expert elicitation (Tables 1 and 2) of Havelaar et al. (2008), which were provided at exposure level, two different methods were applied.

**TABLE 1:** Attribution estimates at the transmission pathway level for *Campylobacter*, nontyphoidal *Salmonella* spp., *Listeria monocytogenes* and STEC O157

Pathogen	Transmission pathway	PAF^a^ (normalized)	Expert estimates, from Havelaar et al. (2008)	Attribution estimates^b^
*Campylobacter* spp.	Food	54.3% (50.9%)	42.0%	56.0% (53.9%–58.2%)
	Environment	17.2% (16.1%)	21.0%	14.6% (13.0%–16.2%)
	Human	4.0% (3.8%)	6.0%	3.2% (2.5%–4.1%)
	Animal	12.9% (12.1%)	19.0%	10.7% (9.4%–12.1%)
	Travel	18.2% (17.1%)	12.0%	15.4% (13.8%–17.0%)
Nontyphoidal *Salmonella* spp.	Food	23.5% (43.2%)	55.0%	46.2% (41.2%–51.0%)
	Environment	N/A	13.0%	5.1% (1.8%–10.2%)
	Human	9.4% (17.4%)	9.0%	11.1% (7.7%–14.9%)
	Animal	4.5% (8.2%)	9.0%	7.6% (4.8%–10.8%)
	Travel	17.0%^c^ (31.2%)	14.0%	30.0% (24.9%–35.4%)
STEC O157	Food	36.9% (64.8%)	40.0%	36.5% (31.5%–42.4%)
	Environment	N/A	17.0%	15.0% (1.8%–36.5%)
	Human	N/A	10.0%	9.6% (1.1%–34.1%)
	Animal	N/A	21.0%	18.4% (2.3%–37.3%)
	Travel	20.0%^c^ (35.2%)	12.0%	20.4% (16.4%–25.0%)
*Listeria monocytogenes*	Food	54.4% (91.6%)	69.0%	78.8% (72.4%–84.2%)
	Environment	N/A	7.0%	5.2% (0.9%–12.8%)
	Human	N/A	5.0%	4.3% (0.7%–11.9%)
	Animal	N/A	5.0%	4.3% (0.6%–11.6%)
	Travel	5.0%^c^ (8.6%)	13.0%	7.3% (4.1%–11.4%)

N/A, not available.

^a^ Population attributable fraction from case‐control studies.

^b^ Ranges within parentheses denote 95% credible intervals (95%CI).

^c^ Obtained from the cases reported to the Dutch national surveillance system for infectious diseases.

**TABLE 2:** Attribution estimates at the food group (i.e. within the foodborne pathway) level for *Campylobacter*, nontyphoidal *Salmonella* spp., *Listeria monocytogenes* and STEC O157

Pathogen	Foodborne exposure	PAF^a^ (normalized)	Expert estimates, from Havelaar et al. (2008)	Attribution estimates^b^
*Campylobacter* spp.	Beef/lamb	11.0% (22.2%)	4.0%	19.5% (16.9%–22.1%)
	Pork	N/A	5.0%	3.1% (0.5%–8.2%)
	Poultry meat	28.0% (54.9%)	54.0%	48.8% (45.5%–52.1%)
	Eggs	N/A	3.0%	2.3% (0.3%–6.9%)
	Dairy	3.0% (5.2%)	9.0%	4.1% (0.8%–9.9%)
	Fish/shellfish/crustaceans	4.0% (7.9%)	7.0%	6.9% (5.3%–8.6%)
	Fruit/vegetables	N/A	5.0%	3.1% (0.5%–8.2%)
	Beverages	N/A	2.0%	1.8% (0.2%–6.0%)
	Grains	N/A	2.0%	1.8% (0.3%–5.7%)
	Composite foods	1.0% (2.0%)	3.0%	1.8% (1.0%–2.7%)
	Food handlers, vermin	4.0% (7.8%)	5.0%	6.9% (5.3%–8.6%)
Nontyphoidal *Salmonella* spp.	Beef/lamb	4.0% (9.8%)	13.0%	7.4% (4.4%–11.1%)
	Pork	14.0% (39.7%)	14.0%	21.9% (14.8%–29.4%)
	Poultry meat	N/A	15.0%	13.2% (3.4%–23.9%)
	Eggs	9.0% (24.4%)	22.0%	15.4% (11.2%–20.0%)
	Dairy	N/A	7.0%	6.8% (1.5%–18.7%)
	Fish/shellfish/crustaceans	N/A	4.0%	4.1% (0.9%–12.7%)
	Fruit/vegetables	N/A	6.0%	5.9% (1.3%–16.9%)
	Beverages	N/A	3.0%	3.2% (0.7%–9.4%)
	Grains	N/A	4.0%	4.1% (0.9%–12.3%)
	Composite foods	N/A	6.0%	5.9% (1.3%–17.5%)
	Food handlers, vermin	9.4% (26.2%)	6.0%	12.3% (8.2%–17.4%)
STEC O157	Beef/lamb	66.9% (87.0%)	44.0%	65.0% (60.1%–69.6%)
	Pork	10.0% (13.0%)	6.0%	9.2% (6.6%–12.1%)
	Poultry meat	N/A	3.0%	2.3% (0.4%–6.9%)
	Eggs	N/A	2.0%	1.8% (0.2%–5.8%)
	Dairy	N/A	7.0%	3.4% (0.6%–9.0%)
	Fish/shellfish/crustaceans	N/A	3.0%	2.3% (0.4%–6.7%)
	Fruit/vegetables	N/A	7.0%	3.5% (0.6%–9.1%)
	Beverages	N/A	4.0%	2.6% (0.4%–7.6%)
	Grains	N/A	3.0%	2.3% (0.3%–7.0%)
	Composite foods	N/A	4.0%	2.6% (0.4%–7.9%)
	Food handlers, vermin	N/A	17.0%	5.1% (1.1%–11.8%)
*Listeria monocytogenes*	Beef/lamb	4.9% (6.9%)	11.0%	6.2% (3.4%–9.8%)
	Pork	4.5% (6.3%)	9.0%	5.6% (2.9%–9.1%)
	Poultry meat	9.3% (13.1%)	7.0%	11.2% (7.3%–15.9%)
	Eggs	N/A	4.0%	2.5% (0.4%–7.5%)
	Dairy	24.4% (34.4%)	25.0%	28.7% (24.6%–33.0%)
	Fish/shellfish/crustaceans	4.6% (6.5%)	18.0%	5.9% (3.1%–9.4%)
	Fruit/vegetables	23.3% (32.8%)	8.0%	28.5% (24.1%–32.6%)
	Beverages	N/A	3.0%	2.2% (0.3%–6.8%)
	Grains	N/A	6.0%	3.2% (0.6%–8.8%)
	Composite foods	N/A	6.0%	3.1% (0.5%–8.8%)
	Food handlers, vermin	N/A	5.0%	2.9% (0.5%–8.2%)

N/A, not available.

^a^ Population attributable fraction from case‐control studies.

^b^ Ranges within parentheses denote 95% confidence intervals (95%CI).

#### 2.2.1 Attribution across reservoirs

For each pathogen, Bayesian statistical modelling was used to estimate the fraction of human cases attributable to each reservoir by accounting for the heterogeneity arising from the use of different typing methods, attribution models and year(s) of data collection in the different studies. This step provided attribution estimates at the reservoir level, which thereby included the contributions of both foodborne and non‐foodborne transmission routes.

For each study *s*, we define *ps_i,s_* as the attribution estimates for each reservoir *i* (with *i = 1*,…,*R*) being *R* the total number of reservoirs. The reservoirs investigated vary among studies (i.e. not all studies considered the same reservoirs), which leads to missing estimates for some reservoirs that are not necessarily zero. Therefore, the values of *ps_i,s_* are partial estimates and would not add up to 1 over all reservoirs in the studies. To circumvent this, we normalized the attribution estimates for all reservoirs by considering the (complete) attribution estimate *p_i,s_* calculated using the following formula:

(1) $$p_{i,s}=\frac{ps_{i,s}}{\sum_{i=1}^{R}ps_{i,s}}$$

to ensure that $\sum_{i=1}^{R}p_{i,s}$ is constrained to 1. This is, in fact, equivalent to using a Dirichlet distribution and a multinomial distribution likelihood. We used the alternative method described above because of the missing data in the dataset, and the Bayesian analysis software JAGS (v4.3) (Plummer, 2003) we used is not able to model a partially observed multinomial distribution.

To ensure that *ps_i,s_* values are naturally bounded between 0 and 1, we introduced the parameter *pp_i,s_*, which relates to *ps_i,s_* by the logistic function

(2) $$ps_{i,s}=e^{pp_{i,s}}/\left(1+e^{pp_{i,s}}\right)$$

The source attribution parameters *pp_i,s_* have prior normal distributions $pp_{i,s}∼N\left(w_{i},e^{z_{i}}\right)$, where *w* and *z* have vague prior normal distributions $w_{i}∼N\left(\mu_{i},\tau_{i}\right)$ and $z_{i}∼N\left(\mu_{i},\tau_{i}\right)$, with mean $\mu_{i}=0$ and precision $\tau_{i}=\left(1/\sigma_{i}^{2}\right)=0.01$ (*σ* is the standard deviation).

The studies providing data for a certain reservoir were heterogeneous in terms of typing methods and types of source attribution models used, as well as years of data collection, and all these factors might influence the attribution estimate *p_i,s_*. To account for this, extra terms were considered: *b_j,s_* defined as the contribution (log‐odds) to the attribution estimate from the various typing methods *j (j = *1,…,*T*; with *T* being the total number of typing methods); *b_k,s_* defined as the contribution (log‐odds) to the attribution estimate from the various source attribution models *k* (*k = *1,…,*M;* with *M* being the total number of source attribution models).

The log‐odds *b_j,s_* and *b_k,s_* followed normal distributions $b_{j,s}∼N\left(m_{j},e^{\epsilon_{j}}\right)$ and $b_{k,s}∼N\left(m_{k},e^{\epsilon_{k}}\right).$ The parameters *m* and ϵ followed vague prior normal distributions, $m_{j}∼N\left(\mu_{j},\tau_{j}\right);\;m_{k}∼N\left(\mu_{k},\tau_{k}\right);$ and $\epsilon_{j}∼N\left(\mu_{j},\tau_{j}\right)$; $\epsilon_{k}∼N\left(\mu_{k},\tau_{k}\right)$; with mean $\mu_{j}=\mu_{k}=0$ and precision $\tau_{j}=\tau_{k}=\left(1/\sigma^{2}\right)=0.01$ (*σ* is the standard deviation).

Then we considered *L* as the total number of unique combinations of *i*, *j, k* and *s*, and we defined the attribution estimate

(3) $$p_{l}=i\text{logit}\left(pp_{i,s}+b_{j,s}+b_{k,s}\right)\;\left(l=1,\ldots ,L\right)$$

where $i\text{logit}\left(x\right)=e^{x}/\left(1+e^{x}\right)$. The estimates were calculated with *L = *54 cases for *Campylobacter*, 110 for *Salmonella*, 128 for *Listeria* and 8 for STEC O157. In the absence of heterogeneities between studies, *p_l_* = *p_i,s_*. For each study‐reservoir‐typing method‐model combination, the number of human cases attributed to each reservoir was modelled as drawn from a binomial distribution:

(4) $${\text{Cases}}_{l}∼\text{Binomial}\left(N_{\text{eff},s},p_{l}\right);\;\left(I=1,\ldots ,L\right)$$

with *N*_eff,s_ being the effective number of human cases in study *s*. As mentioned above, the set of reservoirs investigated varies among studies. Therefore, unless a study covered all possible reservoirs when using a specific typing method and model, there are missing data for each study, and a few extra observations are needed to fill in the unobserved reservoirs in each study. Of note, these extra observations would also increase the precision of the estimates. Consequently, we considered an effective number of human cases *N*_eff,s_ instead of the total observed number of human cases, *N*_total,s_. Specifically, a number of cases *N*_extra,s_ was added to *N*_total,s_ as *N*_eff,s_ = *N*_total,s_ + *N*_extra,s_. *N*_extra,s_ was drawn stochastically for every study *s* from a Poisson distribution *N*_extra,s_ ~Poisson(*λ*_extra,s_), where *λ*_extra,s_, was drawn from a vague prior distribution $\text{Log}\left(\lambda_{\text{extra,s}}\right)∼N\left(0,10\right)$. This allowed the model to fill in the missing reservoirs of each study with a proportion informed by the rest of the studies containing the corresponding reservoirs in question.

Recent studies are more likely to be up‐to‐date and so provide more relevant information for the current situation. Therefore, we gave more weight to the more recent studies by reducing the precision ($\tau =1/\sigma^{2}$) of the older studies as follows:

(5) $${\text{ObservedCases}}_{l}∼N\left({\text{Cases}}_{l},\tau_{\mathrm{hc}}\right),$$

where $\tau_{\mathrm{hc}}=1/{\left(2020‐\text{year}\right)}^{2}$, so if the year is 2020, then $\tau_{\mathrm{hc}}=1$. Thus, Cases*_l_* followed the actual number observed in a study ${\text{ObservedCases}}_{l}$ with increasingly less accuracy according to study's age. We then checked whether the different models and typing methods were predominantly used in older studies, as these studies had less influence on the results. However, even after ignoring the age of the studies, the effects of the model and typing method were minimal.

#### 2.2.2 Attribution across transmission routes

For each pathogen, we combined the PAFs of the significant risk factors from the case‐control studies as to group them, where possible, according to the transmission pathway (Table 1) and the different food groups within the foodborne transmission pathway (Table 2). When PAFs were not reported in the study, we calculated them using Miettinen's formula (Miettinen, 1974) based on the study‐provided multivariable ORs (a proxy for relative risks) and prevalence of exposure among cases, as follows:

(6) $$\text{PAF}=\text{pd}\left[\frac{\left(\text{OR}‐1\right)}{\text{OR}}\right]$$

where pd is the prevalence of exposure to the risk factor among the cases. If necessary (i.e. adjusted ORs or pd not available), Levin's formula (Levin, 1953) was used for the calculation of the PAFs, as follows:

(7) $$\text{PAF}=\frac{\text{pe}\left(\text{OR}‐1\right)}{\text{pe}\left(\text{OR}‐1\right)+1}$$

where pe is the prevalence of exposure to the risk factor in the overall population as obtained from the Dutch National Food Consumption Survey (https://www.rivm.nl/en/dutch‐national‐food‐consumption‐survey) if the risk factors pertained to food consumption, or from a continuous population‐based survey conducted in the Netherlands since 2008 from which the exposure to different risk factors in the general population can be derived (Friesema, van Gageldonk‐Lafeber et al., 2015). This provided attribution estimates at the exposure level for the five transmission pathways and the 11 food groups, to be combined with the estimates from the expert elicitation (Havelaar et al., 2008). For *Salmonella*, STEC O157 and *Listeria*, the fractions attributable to ‘travel’ as transmission pathway could be derived directly from the cases reported to the Dutch national surveillance system for infectious diseases, which are published annually by the Dutch National Institutes for Public Health and the Environment (RIVM) (Vlaanderen et al., 2019).

In a similar way to how the attributions at reservoir level were estimated, a Bayesian framework was used in which prior information is combined with data to arrive at posterior parameter estimates for the fraction of human cases attributable to the different transmission pathways. For the attribution estimates per transmission pathway (also for each food group within the foodborne pathway), there is a total number of cases per study, *N*_total,s_. However, a few of them cannot be classified because the set of studied pathways does not cover all possible existing transmission pathways. Therefore, the number of human cases per pathway reported in a given study *s* that can provide information is *N*_observed,s_, which is less than *N*_total,s_, and consequently reduces the accuracy of a study's estimate *p_q,s_* (with *q* = 1,…, *Q_s_*). Note that *q* denotes the transmission pathway and *Q_s_*, the number of studied transmission pathways in study *s*. Let

(8) $${\text{CP}}_{\text{observed,s}}=1‐\left[\left(1‐p_{1,s}\right)\left(1‐p_{2,s}\right)\ldots \left(1‐p_{\mathrm{Qs}}\right)\right],$$

where CP_observed_ is the cumulative probability of a human case being attributed to one of the transmission pathways observed in study *s* (note that *Q_s_* ≤ *Q*, the total number of possible transmission pathways). Therefore, the total number of cases in study *s* that can be classified into one of the observed transmission pathways can be written as

(9) $$N_{\text{observed,s}}={\text{CP}}_{\text{observed,s}}\times N_{\text{total,s}}$$

To account for all existing pathways, we used a hypothetical effective number of human cases *N*_effective,s_. This effective number is to be used in the stochastic process to correctly calculate the posterior distributions for all pathways, even for those unmeasured in a study. Note that the use of *N*_effective,s_ modifies the precision of the estimates. Based on expert elicitation data (Havelaar et al., 2008), we inferred the total proportion of all transmission pathways observed in a given study *s* by simply adding the expert‐provided fractional values of the pathways observed in the study. For this, we used the point estimates. Let us consider CP_expert,s_, the cumulative proportion (from expert opinions) over all transmission pathways that were observed in study *s*:

(10) $${\text{CP}}_{\text{expert,s}}=p_{1,\text{expert}}+p_{2,\text{expert}}+\cdots +p_{Qs,\text{expert}}.$$

Note that attribution estimates from the expert elicitation study (Havelaar et al., 2008) were normalized to sum to 1. We can then write:

(11) $$N_{\text{observed,s}}={\text{CP}}_{\text{expert,s}}\times N_{\text{effective,s}},$$

which combined with Equation (9) provides the effective number of cases:

(12) $$N_{\text{effective,s}}=\left({\text{CP}}_{\text{observed,s}}/{\text{CP}}_{\text{expert,s}}\right)\times N_{\text{total,s}}$$

The number of human cases observed within transmission pathway *q* in study *s* is specified as binomially distributed, allowing inference on the attribution estimate *p_q,s_*:

(13) $$N_{\text{observed},q,s}∼\text{Binomial}\left(N_{\text{effective,s}},p_{q,s}\right)$$

To allow the data on the proportion for the various pathways from expert opinions, *p_q_*__expert_, to provide information on the missing pathways per study, we assumed that these contribute to the likelihood as data points drawn from log‐normal distributions.

(14) $$\text{Log}\left(p_{q\_\text{expert}}\right)∼N\left(\text{Log}\left(p_{q}\right),\tau_{q\_\text{expert}}\right).$$

Here, *τ_q_*__expert_ is the precision (*τ* = 1/*σ* ^2^) attributed to the logarithm of experts’ fractional estimates, which for this study, we have arbitrarily set to 1, i.e. experts’ fractional estimates were then assumed to be certain by a factor 1.96 × e ≈ 5 up or down.

Calculations were carried out using Markov Chain Monte Carlo (MCMC) sampling. The model was implemented and run in JAGS (v4.3) (Plummer, 2003), interfaced with the statistical language R (v4.03). Five parallel chains were run for 100,000 iterations with a previous burn‐in phase of 10,000 iterations, and convergence was assessed by visual inspection of the posteriors (mixing of chains). The script of the model is available as Data S1.

Ethical approval was not required, as this is a meta‐analytical modelling study synthesizing data from other published studies. Only anonymized, aggregate statistics were available from the primary studies.

## 3 RESULTS

### 3.1 Descriptive results

#### 3.1.1 Studies based on microbial subtyping

Eleven studies based on microbial subtyping passed the screening: 4 Dutch studies on *Campylobacter* (Mughini Gras et al., 2013; Mughini‐Gras et al., 2012, 2020; Smid et al., 2013), three of which used Multilocus Sequence Typing (MLST) data with the Asymmetric Island Model (AIM) (Wilson et al., 2008) and one used core‐genome MLST (cgMLST) data with the STRUCTURE algorithm (Pritchard et al., 2000). These studies provided attributions for a total of 3205 human campylobacteriosis cases (in 2002/2003, 2010/2011 and 2018/2019) to nine reservoirs (broiler chickens, egg‐laying hens, turkeys, beef cattle, dairy cattle, pigs, small ruminants [sheep/goats], dogs/pets and wild birds/environment). For *Salmonella*, three Dutch studies were included (Mughini‐Gras, Enserink, et al., 2014; Mughini‐Gras & van Pelt, 2014; Vlaanderen et al., 2019), two of which used the modified Dutch model (Mughini‐Gras, Smid, et al., 2014) and one used both the modified Dutch and modified Hald (Mullner et al., 2009) models based on serotyping data. These studies provided attributions for a total of 39,174 human salmonellosis cases (during 2002–2018) to five reservoirs (broiler chickens, egg‐laying hens, beef cattle, pigs and reptile pets). For STEC O157, only one Dutch study was available (Mughini‐Gras et al., 2018), which used O‐serotyping data with both the modified Dutch and modified Hald models to attribute 321 human STEC O157 cases (during 2010–2014) to four reservoirs (broiler chickens, beef cattle, pigs and small ruminants). For *Listeria*, there was no Dutch study available on source attribution based on microbial subtyping. Therefore, we included three other European studies, one conducted in the United Kingdom (UK) in 2004–2007 based on sero‐AFLP typing data (Little et al., 2010), one in Northern Italy in 2005–2016 based on MLST and Multi‐Virulence‐Locus Sequence Typing (Filipello et al., 2020) and one in eleven European countries in 2010–2011 using different MLST and cgMLST schemes (Nielsen et al., 2017). These studies provided attributions for 1115 human cases to eight reservoirs (broiler chickens, turkeys, beef cattle, dairy cattle, small ruminants, pigs, fish/shellfish and other/unknown).

#### 3.1.2 Case‐control studies

Five case‐control studies were identified, one for *Campylobacter* (Doorduyn et al., 2010), two for *Salmonella* (Doorduyn et al., 2006; Mughini‐Gras, Enserink, et al., 2014), one for STEC O157 (Mughini‐Gras et al., 2018) and one for *Listeria* (Friesema, Kuiling, et al., 2015). The study on *Campylobacter*, conducted in 2002–2003, included 1315 *C. jejuni* and 121 *C. coli* cases and 3409 frequency‐matched controls and provided PAFs for 18 significant risk factors from multivariable analysis (Doorduyn et al., 2010). The two studies on *Salmonella* were based on comparable data from 2002 to 2003, but one of the two studies (Mughini‐Gras, Enserink, et al., 2014) also included *Salmonella* serotypes other than Typhimurium and Enteritidis, which were the focus of the other study (Doorduyn et al., 2006). In total, they included 414 human cases and 3165 frequency‐matched controls and provided PAFs for 16 significant risk factors from multivariable analysis. The study on STEC O157, conducted in 2010–2014, included 342 STEC cases and 2260 controls and provided ORs (from which PAFs could be derived) for five risk factors from multivariable analysis (Mughini‐Gras et al., 2018). The study on *Listeria* (Friesema, Kuiling, et al., 2015), however, did not identify any significant risk factor other than underlying chronic diseases, so it could not be used for the purposes of source attribution. We therefore used the pooled ORs of a recent systematic review and meta‐analysis of case‐control studies for human listeriosis (Leclercq et al., 2020) to calculate unnormalized PAFs using Levin's formula and the prevalence of exposure *p* obtained from the population‐based surveys mentioned in section 2.2.2.

#### 3.1.3 Attribution estimates at the reservoir level

**FIGURE 1:**
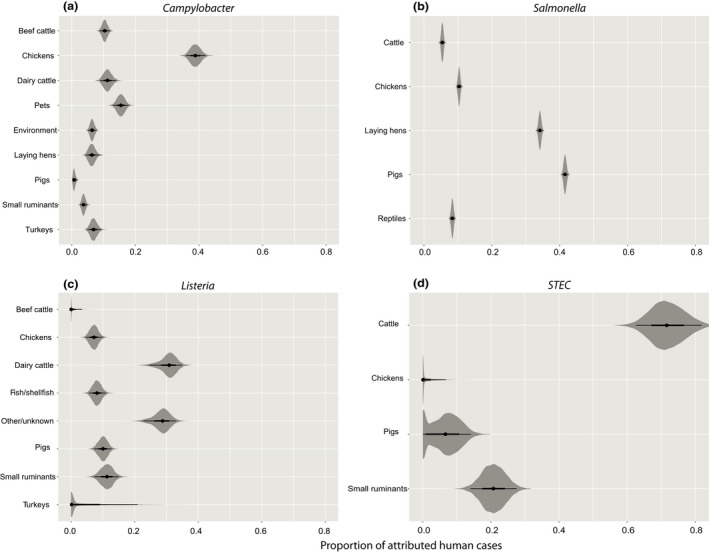
Distribution of the attribution estimates at the reservoir level for (a) *Campylobacter*, (b) nontyphoidal *Salmonella* spp. (c) *Listeria monocytogenes* and (d) STEC O157, based on microbial subtyping data

Figure 1a shows the fractions of human campylobacteriosis cases attributable to the different reservoirs projected by combining the estimates from the four studies based on microbial subtyping. The main reservoir was estimated to be chicken, accounting for 38.9% (95%CI 35.9%–42.0%) of human cases, followed by dogs and cats (15.4%, 95%CI 13.0%–17.8%), beef cattle (10.4%, 95%CI 8.8%–12.0%), dairy cattle (11.2%, 95%CI 8.9%–13.9%), turkeys (6.9%, 95%CI 4.9%–9.1%), laying hens (6.3%, 95%CI 4.4%–8.5%), small ruminants (3.7%, 95%CI 2.7%–4.8%) and pigs (0.8%, 0.2%–1.7%), while 6.4% (95%CI 5.2%–7.7%) of cases was attributed to the environment and other (unknown) sources.

For *Salmonella* (Figure 1b), the most important reservoirs were pigs (41.6%, 95%CI 40.8%–42.4%) and laying hens (34.2%, 95%CI 33.4%–35.0%), followed by chickens (10.4%, 95%CI 9.8%–11.1%), reptile pets (8.4%, 95%CI 7.7%–9.1%) and cattle (5.4%, 95%CI 4.8%–6.1%).

For *Listeria* (Figure 1c), most cases were attributed to dairy cattle (30.6%, 95%CI 24.7%–34.9%), followed by small ruminants (11.1%, 95%CI 7.4%–15.0%), pigs (10.0%, 95CI 7.2%–12.7%), fish/shellfish (8.1%, 95%CI 5.6%–10.9%), chickens (7.2%, 95%CI 4.7%–9.8%), turkeys (3.6%, 95%CI 0.0%–20.9%) and beef cattle (0.6%, 95%CI 0.0%–3.4%), whereas 28.5% (95%CI 22.8%–33.1%) of the cases could not be attributed to any of the sources included in the studies.

For STEC O157 (Figure 1d), cattle were estimated to be the most important reservoir, accounting for 71.9% (95%CI 62.6%–81.8%) of human cases, followed by small ruminants (20.7%, 95%CI 14.0%–27.6%), pigs (6.3%, 95%CI 0.0%–14.1%) and chickens (1.0%, 95%CI 0.0%–6.8%).

Most of the variability in the attributions at reservoir level was accounted for by the typing method for *Campylobacter* and *Listeria*, although generally the variability was limited (Figures S1 and S2).

#### 3.1.4 Attribution estimates for the different transmission pathways

For all the pathogens, the primary transmission pathways were estimated to be food, with attributions ranging from 78.7% (*Listeria*) to 46.2% (*Salmonella*) (Table 1). Travel came next (from 30.0% for *Salmonella* to 7.2% for *Listeria*), followed by the environmental pathway (from 14.6% for *Campylobacter* to 5.3% for *Listeria*). Direct contact with animals appeared to be most important for STEC (18.5%) and person‐to‐person transmission for *Salmonella* (11.1%).

#### 3.1.5 Attribution estimates for the foodborne transmission pathway

Within the foodborne transmission pathway, *Campylobacter* infections were mainly attributed to poultry meat (48.8%) and beef/lamb (19.5%) (Table 2). For *Salmonella*, pork (21.8%), eggs (15.4%) and poultry meat (13.2%) were the main attributable sources of foodborne transmission. Beef/lamb accounted for most of STEC foodborne transmission (65.0%), while dairy products and vegetables accounted for most of foodborne *Listeria* infections (28.7% and 28.5% respectively) (Table 2).

## 4 DISCUSSION

We presented a statistical model within a Bayesian framework to generate attribution estimates, either at the reservoir or transmission pathway level, for sporadic infection with zoonotic pathogens as derived from studies based on microbiological and epidemiological methods. This approach offers the opportunity to blend the results of different (and usually less comprehensive) studies. Individual source attribution studies based on empirical data that provide estimates broken down by reservoir, transmission pathway and (food) subgroups therein at the same resolution as only an expert elicitation can offer, are not available. Our approach therefore provides an alternative to capitalize on the availability of multiple (yet inevitably incomplete) small‐scale studies.

Looking at the attribution estimates, these are biologically plausible. The pathogens can be grouped in several categories with respect to their reservoirs, transmission pathways or food groups. For instance, all four pathogens can be transmitted by multiple pathways, but they are predominantly foodborne, with *Campylobacter* being mostly attributable to the chicken reservoir, *Salmonella* to pigs and *Listeria* and STEC O157 to cattle. Food‐wise, the attributions largely reflected those at reservoir level. For *Salmonella*, about half of the attributions at reservoir level for pigs (41%) and layers (34%) was attributed to the respective food groups, i.e. pork (22%) and eggs (15%), highlighting the role of other sources. For STEC O157, a relatively large contribution of the environmental pathway was estimated (15%). A study on the spatial epidemiology of STEC O157 in the Netherlands (Mulder et al., 2021) found that living in areas with high levels of exposure to small ruminants is associated with increased incidence of human STEC O157 infections, suggesting that environmental exposure to small ruminants is a significant risk factor for STEC O157. Also, for *Campylobacter*, a relatively large attribution to the environmental transmission pathway was estimated (15%). Contamination of surface water with *Campylobacter* is widespread in the Netherlands and mostly concerns wild bird‐associated strains (Mulder et al., 2020). However, the contribution of poultry to surface water contamination with *Campylobacter* has been found to be associated with the magnitude of poultry production (Mughini‐Gras, Penny, et al., 2016; Mulder et al., 2020).

While results may be expected to vary over countries due to differences in food production and consumption patterns, these estimates are consistent with the general knowledge on these pathogens. Except perhaps for *Listeria,* which calculations were based on data from other European countries, our results pertain to the Netherlands and should not be expected to be like those in other countries, especially countries outside Europe characterized by the frequent occurrence of foodborne outbreaks linked to products that are hardly ever implicated as sources in the Netherlands and other European countries, such as vegetables and grains among others. This is, for example, the case of the USA where generally larger numbers of foodborne outbreaks are reported, most of which being linked to fresh produce (https://www.cdc.gov/foodsafety/outbreaks/multistate‐outbreaks/outbreaks‐list.html). Accordingly, in the USA, experts tend to attribute a far greater role to produce (Hoffmann et al., 2007). The attributions to some ‘rare’ sources tended to shrink as compared to the expert estimates, as these estimates were probably influenced by the considerable (media) attention that outbreaks usually receive, whereas the attributions reported here pertain to the sporadic cases, which are less likely to make the news. An example are the sources that experts are inclined to consider important due to some outbreaks that sparkled interest in past years, such as some campylobacteriosis outbreaks linked to unpasteurized dairy (Heuvelink et al., 2009; Kenyon et al., 2020; MMWR, 2013), although it is now clearer that campylobacteriosis is mostly sporadic and that those outbreaks were more an exception than a rule. That is probably why the attribution for *Campylobacter* to dairy decreased compared to the expert elicitation.

Our approach was also able to capture trends in the attributions by downweighing the importance of older studies. This is exemplified by the attributions of *Salmonella* to pork and eggs at the exposure level. Indeed, the expert elicitation was performed in a period (2008) in which the most frequently isolated *Salmonella* serovar from human cases was Enteritidis, a serovar strongly associated with laying hens (Mughini‐Gras, Smid, et al., 2014) and therefore mostly transmitted with eggs (Doorduyn et al., 2006), followed by serovar Typhimurium, associated with pigs (Mughini‐Gras, Smid, et al., 2014). However, over the years, Enteritidis has decreased spectacularly in the Netherlands and other European countries, whereas Typhimurium and its monophasic variant have increased (Mughini‐Gras, Heck, et al., 2016; Mughini‐Gras, Smid, et al., 2014). Since 2011, Enteritidis is no longer the primary serovar, meaning that laying hens are nowadays no longer the primary reservoir and that pigs have taken the first place. Therefore, the attribution estimates now better reflect the current situation. In general, the synthetized attribution estimates were more similar to those of the original studies than the expert elicitation. This was particularly evident for the *Campylobacter* attribution to food as transmission pathway, which increased in importance, particularly for beef/lamb as food product. Accordingly, recent studies have highlighted a more prominent role of cattle as source of human campylobacteriosis (Mughini‐Gras et al., 2020; Thepault et al., 2018). For *Salmonella*, the contribution of travel gained importance as compared to expert estimates, which agrees in magnitude with recent findings of a significant drop in notified (travel‐related) salmonellosis cases during the COVID‐19 pandemic as a result of travel restrictions (Mughini‐Gras et al., 2021). Conversely, *Listeria* attribution to travel decreased compared to expert estimates, which seems to better reflect the reality since contrary to salmonellosis, listeriosis did not seem to be affected by travel restrictions during the COVID‐19 pandemic (Benincà et al., 2021).

The attributions are estimated separately for the reservoirs, the transmission pathways, and the exposures within the foodborne pathway; thus, they provide separate pictures of the relative contributions of different sources at defined points in the transmission chain, without inferring a (quantitative) relationship between these points. Another limitation is intrinsic in the datasets used, as no empirical study is as comprehensive and complete (in terms of number and resolution of the sources) as an expert elicitation. This means that for some sources, only the estimates from expert elicitations are available and cannot be updated with other studies unless these become available in the future. Although our method can also be used with other types of data (e.g. from original studies, unpublished material, surveys, etc.) than data obtained from the literature alone, and a literature review was not in itself the goal of this study, the type and quality of the literature search (or data collection in general) will define the interpretation of the model outcomes. The method presented here may thus be used in other settings (i.e. studies), provided that the necessary data are collected from the literature or other sources of information in a way that suits the scope of the study in question. Indeed, the data collection phase must be tailored to the specific research question, not the method per se, and cannot therefore be taken as such from the present study. Yet, different research questions can be answered with the method presented here. While the literature search of this study is not generalizable to other situations as it was meant to fulfil the needs of a specific goal (i.e. model development with application to four major foodborne pathogens in the Netherlands), it can be amended for the purposes of other studies. The same is true for the way the attribution estimates are presented. For example, for the attributions at the exposure level, we used the source categorization defined by the expert elicitation available in the country (Havelaar et al., 2008) because this offered the most complete overview of source categories and corresponding attribution estimates. However, this could be different in another setting where it is possible that this categorization is inapplicable or the focus of the study is limited to, e.g. the food‐related sources. In that case, both the data collection phase and the framework (i.e. categorization of sources) will have to be amended.

In conclusion, we developed a meta‐analytical model that combines the attribution results from different studies and provides robust, biologically plausible estimates, being a valid solution to reach consensus estimates reflecting the available empirical evidence as to inform policy.

## CONFLICT OF INTEREST

The authors declare that there is no conflict of interest.

## Supporting information

Figures S1‐S2

Data S1
