# Supplementary material for: A statistical modelling approach for source attribution meta‐analysis of sporadic infection with foodborne pathogens
Source: Zoonoses Public Health. 2022 Mar 10;69(5):475–86. doi: 10.1111/zph.12937 (PMC9545847; doi:10.1111/zph.12937)

**Supplementary Information**

**Supplementary Figure 1.** Contribution (log-odds) of the different source attribution models (*b_k_*) to the attribution estimates of human infections with *Campylobacter*, nontyphoidal *Salmonella* spp., *Listeria monocytogenes*, and STEC O157 in the. For each pathogen, the upmost model shown is arbitrarily considered as reference, therefore, it shows 0 contribution.


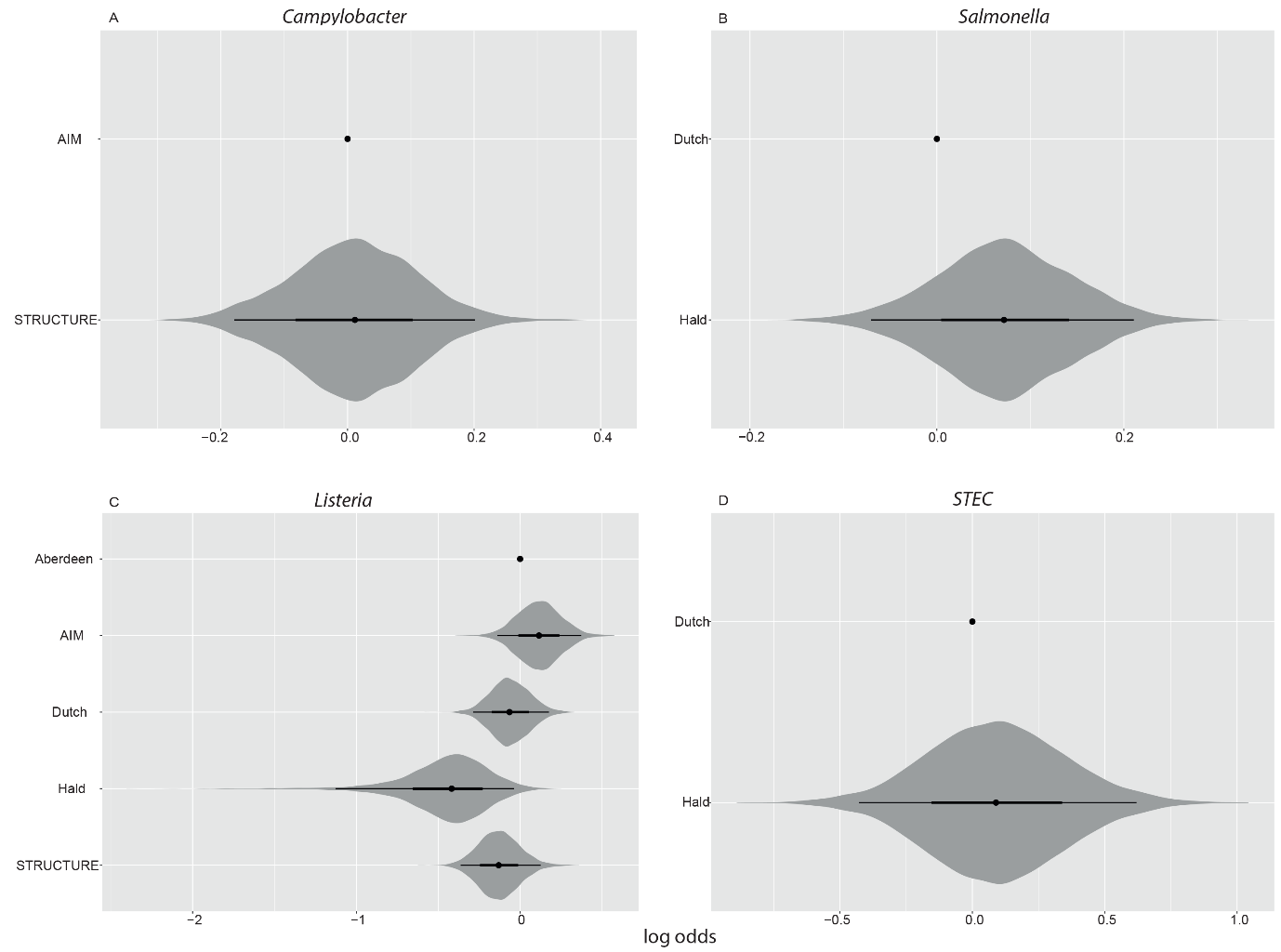


**Supplementary Figure 2.** Contribution (log-odds) of the different typing methods (*b_j_*) to the attribution estimates of human infections with *Campylobacter* and *Listeria monocytogenes* in the Netherlands. For each pathogen, the upmost model shown is arbitrarily considered as reference, therefore, it shows 0 contribution.


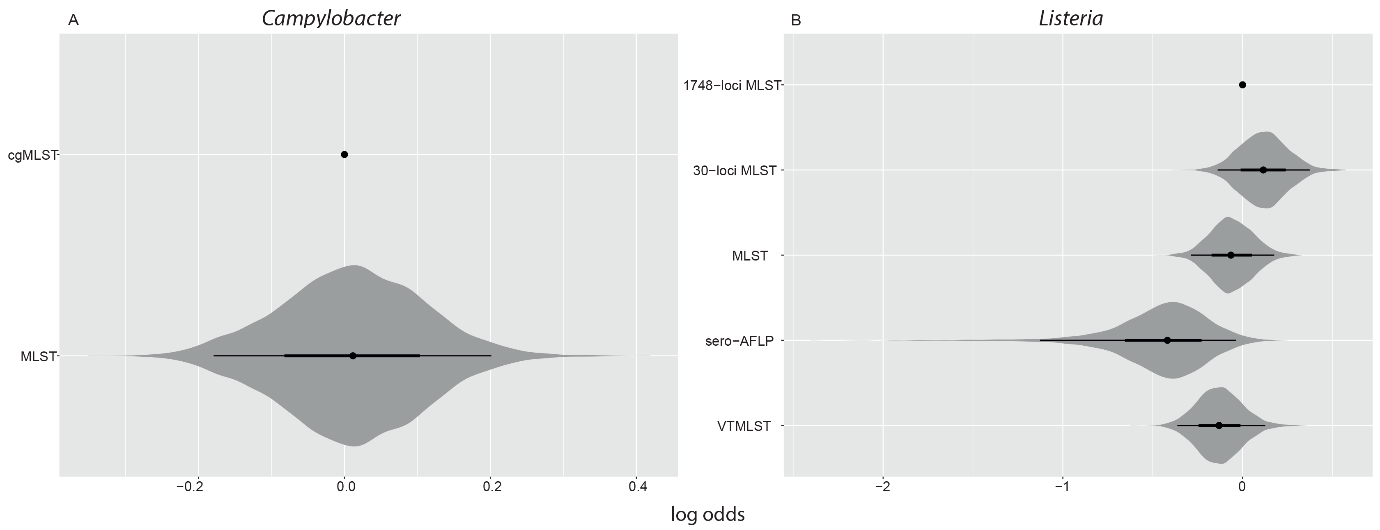

Supplement: Supplementary file 1 — Figures S1‐S2 [file ZPH-69-475-s001.docx]
